# Gene expression signatures and cardiometabolic outcomes following 8-week mango consumption in individuals with overweight/obesity

**DOI:** 10.3389/fnut.2022.918844

**Published:** 2022-08-11

**Authors:** Justine Keathley, Juan de Toro-Martín, Michèle Kearney, Véronique Garneau, Geneviève Pilon, Patrick Couture, André Marette, Marie-Claude Vohl, Charles Couillard

**Affiliations:** ^1^Center Nutrition, Santé et Société (NUTRISS)-Institut sur la Nutrition et les Aliments Fonctionnels (INAF), Université Laval, Québec, QC, Canada; ^2^School of Nutrition, Université Laval, Québec, QC, Canada; ^3^Quebec Heart and Lung Institute (IUCPQ) Research Center, Québec, QC, Canada; ^4^Endocrinology and Nephrology Unit, CHU de Quebec Research Center, Québec, QC, Canada

**Keywords:** transcriptomics, cardiometabolic risk factors, precision nutrition, mango, *Mangifera*

## Abstract

**Background:**

Little is known about the impact of mango consumption on metabolic pathways assessed by changes in gene expression.

**Methods:**

In this single-arm clinical trial, cardiometabolic outcomes and gene expression levels in whole blood samples from 26 men and women were examined at baseline and after 8 weeks of mango consumption and differential gene expression changes were determined. Based on changes in gene expression profiles, partial least squares discriminant analysis followed by hierarchical clustering were used to classify participants into subgroups of response and differences in gene expression changes and in cardiometabolic clinical outcomes following the intervention were tested.

**Results:**

Two subgroups of participants were separated based on the resemblance of gene expression profiles in response to the intervention and as responders (*n* = 8) and non-responders (*n* = 18). A total of 280 transcripts were significantly up-regulated and 603 transcripts down-regulated following the intervention in responders, as compared to non-responders. Several metabolic pathways, mainly related to oxygen and carbon dioxide transport as well as oxidative stress, were found to be significantly enriched with differentially expressed genes. In addition, significantly beneficial changes in hip and waist circumference, c-reactive protein, HOMA-IR and QUICKI indices were observed in responders *vs.* non-responders, following the intervention.

**Conclusion:**

The impact of mango consumption on cardiometabolic health appears to largely rely on interindividual variability. The novel transcriptomic-based clustering analysis used herein can provide insights for future research focused on unveiling the origins of heterogeneous responses to dietary interventions.

**Clinical Trial Registration:**

[clinicaltrials.gov], identifier [NCT03825276].

## Introduction

The transcriptome is a set of RNA species, which are transcribed at a cellular level and contribute to physiological functioning, as well as disease progression and protection (1). Cells express different sets of genes in response to environmental exposures (2). For example, certain dietary components can promote human health through transcriptomic modulation leading to differences in gene expression (3–5). Variations in gene expression resulting from certain environmental exposures, such as dietary intake, can provide important mechanistic insights on the impact of these exposures on human health and disease. Moreover, transcriptomics can help identify new therapeutic targets relevant to numerous conditions (2).

Mango *(Mangifera indica)* is a fruit that contains high amounts of phenolic compounds including mangiferin, gallotannins and phenolic acids (6). Mangos are also good sources of carbohydrates, dietary fiber, vitamin C, vitamin A (beta-carotene) and potassium (7). Moreover, this fruit is widely consumed globally, ranking fourth on the list of total production for major fruit crops around the world (8). With mangos containing several beneficial bioactive components, a number of health benefits have been associated with mango consumption including improvements in blood pressure and blood glucose levels, as well as reductions in diabetes and cardiovascular disease risk, among others (9–11). While research has demonstrated the impact of mango consumption on health outcomes, the potential underlying mechanisms remains poorly understood. Additionally, to our knowledge, a transcriptomic approach has never been used to identify pathways and potential molecular mechanisms for the impact of mango consumption on human health. Therefore, this study aimed to explore the mechanisms of action of mango consumption on cardiometabolic outcomes through transcriptomic analyses.

## Materials and methods

### Participants

This study was approved by the Ethics Committee of [removed for blind peer review] and was registered with clinicaltrials.gov (NCT03825276). In this exploratory, single-arm clinical trial, participants were recruited through social media advertisements as well as from email listings of students and employees of Université Laval and study recruitment list from the [removed for blind peer review]. Inclusion criteria consisted of men and women of Caucasian ancestry 18–55 years of age (premenopausal for women), with a body mass index (BMI) ≥ 25 kg/m^2^ and ≤ 40 kg/m^2^, and a waist circumference ≥ 80 cm for women and ≥ 94 cm for men (or one of these two in addition to insulin > 42 pmol/L or triglycerides > 1.35 mmol/L). Participants were excluded if they: were nicotine users; had consumed dietary supplements or natural health products that could affect study outcomes or antibiotics in the previous 3 months; had been diagnosed with a condition or took medication that could affect study outcomes; had surgery over the previous 3 months or planned during the study timeline; consumed > 2 standard alcoholic beverages/day; experienced > 5% weight change over the previous 3 months; consumed > 1 serving of berries and/or mango daily; had an aversion, allergy or intolerance to mango; followed a restricted diet such as vegetarianism or gluten-free; or were pregnant, breastfeeding or planned on becoming pregnant in the following 3–6 months. Each participant received a health report and financial compensation for their participation in the study. Socio-demographic information and medical/familial histories of diseases were collected at baseline.

### Intervention

Participants were instructed to consume 280 g/day (2 cups/day) of frozen mango pulp for 8 weeks following a 2-week run-in period. Mangos were provided to participants and a registered dietitian helped participants incorporate them into their diet while minimizing the impact on their usual food and calorie intakes. During the run-in and treatment periods, participants were instructed to avoid changes in dietary supplements and natural health products; consume a maximum of 2 supplemental servings/week of mango or berry containing foods; consume a maximum of 1 serving/day of tea or 4 servings/day of coffee; consume a maximum of 2 servings/week of food/beverages containing cocoa and consume no more than 2 standard alcoholic drinks weekly, with red wine being prohibited. Participants were also asked to maintain their body weight and physical activity level. Physical activity level was measured using the Leisure Time Activity questionnaire (12) to assess compliance. To assess if there were any significant changes in nutritional intake throughout the duration of the study, participants completed a validated self-administered online food frequency questionnaire (13).

### RNA sequencing

The effects of mango consumption on gene expression profiles were examined in whole blood samples of study participants. Fasting blood samples were collected into PAXgene^®^ preparation tubes (Qiagen, Valencia, CA, United States) at week 0 and 8. Total RNA was extracted with the RNeasy Mini Kit (Qiagen, Valencia, CA, United States) and quality evaluated with the 2100 Bioanalyzer (Agilent, Santa Clara, CA, United States). RNA sequencing was performed at the *Centre d’expertise et de services Génome Québec*. Briefly, library preparation was carried out using the Illumina NEB stranded mRNA library preparation kit (Illumina, San Diego, CA, United States) and sequencing was performed on the Illumina NovaSeq6000 S4 platform (Illumina) using 100 bp paired-end reads. Raw reads were first trimmed at 50 bases and at a Phred quality score of 30 using Trim Galore (v0.6.5) (14). Read quantification was performed using kallisto (v0.46.2) (15) with 100 bootstraps and reads were aligned to the GRCh38 human reference transcriptome. The obtained transcript counts were filtered by expression level and normalized by TMM (trimmed mean of M-values).

### Discriminant analysis and hierarchical clustering

The identification of sub-groups of participants was performed by multilevel partial least squares discriminant analysis (PLS-DA) and hierarchical clustering analysis (HCA). First, the most variable transcripts between week 0 and week 8 in the entire group of participants at nominal *p*-value < 0.05 were used as input data for PLS-DA. Second, the two main components resulting from PLS-DA were used as input for HCA with Euclidean distance and Ward linkage criterion to identify relevant sub-groups of participants. HCA was performed in the pvclust R package, which computes approximately unbiased *p*-values (AU) for each of the clusters *via* multiscale bootstrap resampling (*n* = 1,000 replications). Cluster characterization was based on the homogeneity of participants, the directionality of time points (before or after the intervention) and the discriminative power of gene expression profiles. A sparse PLS-DA (sPLS-DA), with 10-fold cross validation and repeated 10 times, was finally used to maximize the sub-group discrimination and to identify the most relevant transcripts that help discriminate newly identified sub-groups. Discriminant analysis algorithms were implemented using the mixOmics R package (v6.12.1) (16), which was also used for outlier detection by multilevel principal component analysis (PCA). Concretely, we used a modified version of the six standard deviation from the mean method and implemented in the bigutilsr R package (v0.3.4).

### Differential expression analysis

After clustering analysis, differences in gene expression changes following the intervention were tested between sub-groups and significant transcripts were considered at a false-discovery rate (FDR)-corrected *p*-value < 0.05 and showing at least a 50% difference (1.5-fold change) between sub-groups. Differential gene expression was determined using a generalization of a *t*-test implemented in the quasi-likelihood framework of edgeR (v3.28.1) (17). In order to take into account baseline differences between participants, differential gene expression analysis was performed by using a paired design. The functional roles and relationships of significant genes were explored by pathway enrichment analysis using the clusterProfiler (v3.16.0) R package (18). The Gene Ontology Biological Processes (GO-BP) and the Reactome pathway databases were used for functional enrichment analysis. Differentially expressed genes, as well as most relevant genes identified in the sPLS-DA model, were used as input data.

### Assessment of cardiometabolic outcomes

At each visit to the clinical investigation unit at [removed for blind peer review], height, waist and hip circumferences were measured to the nearest mm. Body weight was measured using a BWB-800 electronic scale (Tanita, Arlington Heights, IL, United States) to an accuracy of 0.1 kg, with participants wearing light indoor clothes without shoes. BMI was calculated as weight in kg divided by height in meters squared (kg/m^2^). Systolic and diastolic blood pressures (BP) were assessed while participants were seated after a 10-min rest. The mean of three measurements performed at 3-min intervals was used for the analyses. Blood samples were collected in the morning, after a 12-h fast. Plasma total cholesterol, triglyceride and HDL-cholesterol concentrations were assessed on a Siemens Dimension Vista 1500. Serum LDL-cholesterol concentrations were calculated with the Friedewald equation (19). Plasma high sensitivity C-reactive protein (hs-CRP) concentrations were determined with the Behring Latex-Enhanced highly sensitive assay on a Behring Nephelometer BN-100 (Behring Diagnostics). Oral glucose tolerance tests (OGTTs) using a 75-g glucose solution, were conducted twice during the intervention period (weeks 0 and 8). Blood samples were drawn fasted and 2 h following glucose ingestion. Glucose concentrations were measured by enzymatic assay (Siemens Dimension Vista 1500) and insulin concentrations were assessed on a Siemens Dimension Vista 1500. Fasting glycated hemoglobin (HbA1c) was measured in plasma by ion-exchange HPLC (Biorad D-100) and insulin resistance was calculated with the homeostatic model assessment of insulin resistance (HOMA-IR) formula (20), composite Matsuda index (21), and quantitative insulin sensitivity check index (QUICKI) (22).

### Statistical analyses of cardiometabolic outcomes

Statistical analyses for the clinical outcomes included linear mixed models using SPSS version 26.0. Age, sex and BMI were included as covariates in the model, except for the outcomes of BMI, waist circumference and hip circumference in which only age and sex were included as covariates. A *p*-value < 0.05 was considered statistically significant.

## Results

### Discriminant analysis and hierarchical clustering

**FIGURE 1:**
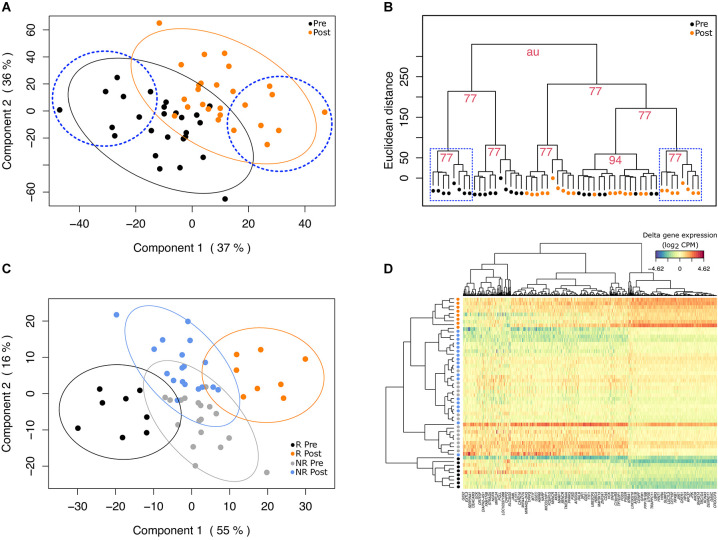
Discriminant analysis and hierarchical clustering. **(A)** Projection of pre- and post-intervention visits (black and orange dots, respectively) of the ungrouped cohort onto the space, spanned by the two principal components derived from partial least-squares discriminant analysis (PLS-DA). The two first components of the model along with their corresponding explained variance are shown on x- and y-axis, respectively. Ellipses represent 95% confidence intervals around the centroid of each visit group. Blue dashed circles encompass participants into the newly sub-group of responders identified by hierarchical clustering analysis (HCA). **(B)** Dendrogram resulting from applying HCA (Ward’s method) on the two first components of PLS-DA. The Euclidean distance on the y-axis measures the dissimilarity between each pair of observations. Blue dashed squares encompass matched pre- (black dots) and post-mango consumption visits (orange dots) of participants from the so-called responder sub-group. Numbers represent approximately unbiased *p*-values (AU) for each cluster. **(C)** Results from multilevel sPLS-DA show the complete discrimination between pre- (black dots) and post- visits (orange dots) in the responder (R) group. In the non-responder group (NR), pre- (gray dots) and post- visits (blue dots) are mixed. The two first components of the sPLS-DA accounted for 55% and 16%, and were composed of 230 and 460 genes, respectively. **(D)** The heatmap shows group classification (26 matched-pairs in rows) based on the two main sPLS-DA components (genes in columns). Within the group of responders, pre- (black dots) and post-mango consumption visits (orange dots) are clearly separated on the top and bottom rows of the heatmap.

After transcript filtering and normalization, one participant was excluded by outlier detection with multilevel PCA (Supplementary Figure 1), resulting in 26 participants for further discriminant analysis. A total of 1,549 transcripts were identified as being the most variable over the 8-week intervention at a nominal *p*-value < 0.05 and were used as input in the PLS-DA. Results from the multilevel PLS-DA showed that the first two components explained 37 and 36% of variance, respectively (Figure 1A). We used these two first components based on the performance results of the PLS-DA model (Supplementary Figure 2). With these two components used as input, HCA revealed two clusters of matched participants with homogenous and well-discriminated (AU > 75%) pre- and post-intervention visits, composed of 8 participants each (Figure 1B). These two clusters were regrouped into a new subgroup of participants that were re-coded as responders (i.e., showing resemblance in gene expression profiles, *n* = 8) while the rest of participants were identified as non-responders (i.e., showing heterogeneity in expression profiles, *n* = 18). Further, sPLS-DA was used to maximize cluster resolution and to determine transcripts contributing the most to subgroup discrimination. The two first gene expression components of the sPLS-DA model together explained 71% of variance, with a good discrimination in gene expression between pre- and post-mango consumption in the responder sub-group (Figure 1C). By contrast, in the non-responder group, gene expression was not able to discriminate between pre- and post-mango consumption visits. Components 1 and 2 were composed of 230 and 460 transcripts (Figure 1D), respectively, as derived from sPLS-DA tuning process (Supplementary Figure 3). According to a pathway enrichment analysis based on GO-BP terms, most of genes included in component 1 were mainly related to catabolic and metabolic processes, as well as to hydrogen peroxide metabolism (Supplementary Figure 4). By contrast, genes include in component 2 were associated to immune and inflammatory biological processes, as well as with protease activity (Supplementary Figure 4).

### Differential gene expression between sub-groups

**FIGURE 2:**
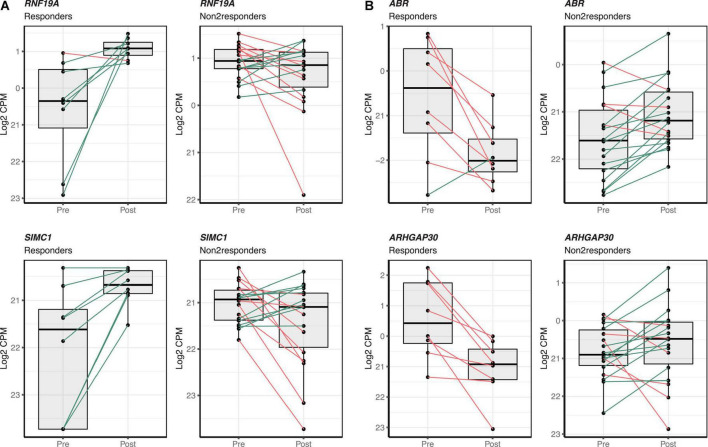
Top differentially expressed transcripts in the responder sub-group following the mango consumption. Individual gene expression change between pre- and post-mango consumption visits is shown for responder and non-responder groups. **(A)** The two transcripts showing the most significant over-expression in the group of responders (*RNF19A* and *SIMC1*) are shown on the two left columns. **(B)** The two transcripts showing the most significant under-expression (*ABR* and *ARHGAP30*) are shown on the two right columns. Box and whisker plots show median, first, and third quartiles, and maximum and minimum values for the 26 matched participants before (Pre) and after (Post) the mango consumption. Green and red lines stand for increasing or decreasing gene expression levels between pre- and post-mango consumption visits within individual paired samples.

Differences in gene expression changes following the intervention were tested between responders and non-responders. After adjustments for multiple testing, at FDR-adjusted *p*-value < 0.05 and a fold change between groups higher than 1.5, a total of 280 transcripts were significantly up-regulated and 603 significantly down-regulated following the intervention in responders. The top 100 up- and down-regulated transcripts in the group of responders can be accessed in Supplementary Table 1. Up- and down-regulated transcripts in the responder group tended to show a similar pattern; this was not observed in the non-responders (Figure 2). *RNF19A and SIMC1* were the most significantly over-expressed in responders whereas *ABR* and *ARHGAP30* were the most significantly under-expressed in responders (Figure 2). Functional enrichment analysis was performed with the 883 differentially expressed transcripts in responders as input. Several pathways were found as significantly enriched at FDR-adjusted *p*-value < 0.05 in the GO-BP and Reactome pathway databases (Supplementary Figure 5).

### Effect of clustering on cardiometabolic clinical outcomes

Participants included in the present study (*n* = 26) were young adults (mean age 29.3 ± 7.7) who were primarily female (*n* = 19). While the mean baseline BMI was greater than 30.0 kg/m^2^, cardiometabolic profiles demonstrated that the participants were otherwise generally healthy. The clustering of participants based on changes in gene expression directly related to the cardiometabolic responses to mango consumption. There were several significant interactions between the responder/non-responder status and time (Table 1). Such interactions were observed for hip and waist circumferences, which were reduced in responders while they were increased in non-responders at the end of the intervention. For hs-CRP, responders demonstrated a reduction after the intervention, with non-responders demonstrating increases. In addition, HOMA-IR decreased in responders, yet increased in non-responders, and vice versa for QUICKI. Notably, the direction of changes for all of the abovementioned significant interactions were in favor of healthier response profiles to the intervention in responders (Table 1). Finally, irrespective of the responder/non-responder status of participants, we noted a time effect of mango consumption on blood pressure and 2-h glucose, which were significantly reduced following the intervention (Table 1).

**TABLE 1:** Cardiometabolic outcomes over time within and between groups.

	Responders (*n* = 8)		Non-responders (*n* = 18)		*P*-values		
Variable	Week 0	Week 8	Week 0	Week 8	Group	Time	Group × Time
BMI (kg/m^2^)	30.8 ± 1.2	30.7 ± 1.3	30.2 ± 0.8	30.3 ± 0.8	0.78	0.39	0.18
Waist circumference (cm)	100.8 ± 3.0	99.5 ± 3.2	95.1 ± 1.9	96.1 ± 2.0	0.23	0.76	**0.002**
Hip circumference (cm)	110.8 ± 2.2	110.0 ± 2.5	110.7 ± 1.4	111.4 ± 1.6	0.82	0.78	**0.02**
Systolic BP (mmHg)	115.7 ± 2.1	112.2 ± 2.4	117.0 ± 1.3	112.7 ± 1.5	0.72	**0.003**	0.74
Diastolic BP (mmHg)	74.0 ± 2.5	71.8 ± 2.1	75.8 ± 1.6	72.4 ± 1.3	0.66	**0.01**	0.43
Total-C (mmol/L)	4.6 ± 0.29	4.79 ± 0.29	4.83 ± 0.19	4.84 ± 0.18	0.73	0.32	0.39
Triglycerides (mmol/L)	1.00 ± 0.25	1.12 ± 0.25	1.50 ± 0.16	1.56 ± 0.16	0.26	0.88	0.83
HDL-C (mmol/L)	1.46 ± 0.11	1.40 ± 0.13	1.43 ± 0.07	1.48 ± 0.08	0.84	0.90	0.17
LDL-C (mmol/L)	2.72 ± 0.26	2.88 ± 0.24	2.70 ± 0.17	2.64 ± 0.16	0.66	0.50	0.15
Total-C: HDL-C	3.41 ± 0.35	3.62 ± 0.42	3.60 ± 0.42	3.63 ± 0.27	0.96	0.23	0.09
LPS (ng/mL)	81.3 ± 21.91	75.5 ± 17.9	112.2 ± 14.0	94.8 ± 11.4	0.37	0.52	0.47
LBP (μg/mL)	16.0 ± 2.47	10.8 ± 1.5	13.1 ± 1.6	14.5 ± 0.9	0.37	0.59	0.07
Creatinine (μmol/L)	69.9 ± 2.6	69.0 ± 2.4	71.3 ± 1.6	70.0 ± 1.5	0.65	0.44	0.90
HbA1C (%)	4.91 ± 0.01	4.90 ± 0.01	5.00 ± 0.00	5.01 ± 0.01	0.39	0.90	0.85
hs-CRP (mg/L)	0.22 ± 0.16	0.07 ± 0.15	0.24 ± 0.10	0.30 ± 0.10	0.49	0.18	**0.007**
Fasting glucose (mmol/L)	4.77 ± 0.15	4.75 ± 0.12	4.82 ± 0.09	4.85 ± 0.07	0.62	0.94	0.68
Fasting insulin (pmol/L)	107.5 ± 10.96	103.0 ± 14.4	72.6 ± 7.0	93.4 ± 9.2	0.12	0.26	0.09
2-hr glucose (mmol/L)	5.12 ± 0.38	4.61 ± 0.35	5.81 ± 0.25	5.18 ± 0.22	0.12	**0.02**	0.91
2-hr insulin (pmol/L)	546.1 ± 91.9	536.6 ± 93.7	475.5 ± 58.2	449.8 ± 58.0	0.40	0.40	0.65
C-peptide (mmol/L)	2404.3 ± 240.7	2375.0 ± 255.2	2271.8 ± 153.4	2217.4 ± 162.8	0.60	0.74	0.92
HOMA-IR	3.80 ± 0.41	3.61 ± 0.60	2.61 ± 0.26	3.47 ± 0.38	0.33	0.44	**0.045**
Matsuda index	3.36 ± 0.76	4.61 ± 0.85	4.39 ± 0.48	4.27 ± 0.53	0.54	0.43	0.21
QUICKI	0.32 ± 0.01	0.33 ± 0.01	0.34 ± 0.01	0.33 ± 0.01	0.39	0.55	**0.04**

Data are means ± standard error. Age, sex and BMI were included as covariates in the model for all variables except BMI, waist circumference and hip circumference in which only age and sex were covariates. A linear mixed model was used to obtain *p*-values. Time effect, responder/non-responder effect and the interaction of responder/non-responder by time were significant at α = 0.05. Significant *p*-values are in bold. The following non-normally distributed variables were transformed to achieve normality: total-C: HDL-C ratio, hs-CRP, LPS, LBP, triglycerides, HOMA-IR, diastolic BP, Matsuda index, 2-hr insulin and 2-hr glucose. BP, blood pressure; HDL-C, high-density lipoprotein cholesterol; LDL-C, low-density lipoprotein cholesterol; QUICKI, quantitative insulin sensitivity check index; HOMA-IR, homeostatic model assessment of insulin resistance; HbA1C, hemoglobin A1C; hs-CRP, high-sensitivity C-reactive protein; LPS, lipopolysaccharide; LBP, lipopolysaccharide binding protein.

## Discussion

The main finding of the present study showed that participants could be classified in two subgroups (responders *vs.* non-responders) based on of similarities of the changes in gene expression profiles in response to mango consumption. It was further interesting to observe that there were significant time × subgroup interactions for the cardiometabolic effects of mango consumption, whereby responders demonstrated improvements in waist and hip circumferences, hsCRP concentrations as well as HOMA-IR and QUICKI indices. These results suggest that the transcriptomic-based clustering may have physiological relevance and pointed to a latent interindividual variability that seems to underly the heterogeneous impact of mango consumption on metabolic health. Results of the present study are primarily generalizable to young, Caucasian adults with overweight and obesity. Future research should first validate our results and further explore the health impacts of mangos using a multi-omics approach. To our knowledge, this was the first study to use a transcriptomics approach to explore the impact of mango pulp consumption on metabolic pathways.

The transcriptomic results that we report herein provide insights to identify new metabolic pathways affected by mango consumption, in individuals who responded to the intervention. These pathways include hydrogen peroxide catabolic and metabolic processes, cofactor catabolic processes, gas transport, oxygen transport, take up of carbon dioxide and release of oxygen by erythrocytes, oxygen and carbon dioxide exchange in erythrocytes, as well as transcriptional regulation by RUNX1 and by TP53. Hydrogen peroxide metabolism has been demonstrated to impact biological processes related to insulin resistance and diabetes, cardiovascular disease, cancer and kidney disease (23). Previous studies with mango extracts have demonstrated beneficial health effects related to these conditions (9, 10, 24). Moreover, in the responder group of the present study, there were several up- and down-regulated genes that are involved in physiological processes related to these conditions such as *TNFAIP3, API5*, and *TAL1* among others. Based on these mechanistic findings, in addition to our findings of changes in waist and hip circumference, hs-CRP, HOMA-IR and QUICKI among responders, it appears that mango consumption could have beneficial cardiometabolic effects that may be predicted by clustered gene expression profiles. Interestingly, while genes included in the first component of the sPLS-DA model were also related to the biological processes enriched with differentially expressed genes, those in the second component were associated to immune and inflammatory pathways, demonstrating the many facets of the metabolic response to mango consumption. Future research should explore if and how the health outcomes noted following mango consumption may be related to these metabolic pathways. However, not all cardiometabolic improvements appear to be connected to similarities in gene expression responses to mango consumption as we noted a significant reduction in blood pressure, which was independent of the gene expression clustering approach. Of note, this reduction in blood pressure following mango consumption is concordant with previous observations (11).

On the other hand, the metabolic pathways modulated by mango consumption could also be used to explore potential physiological effects of the latter. For example, RUNX1 has been demonstrated to be one of the most frequently mutated genes in several hematological malignancies (25). Moreover, research demonstrates that TP53 is the most frequently expressed protein variant in human carcinoma (26). Previous research has also suggested that, at a physiological level, mangiferin modulates key inflammatory pathways involved in cancer progression (10, 27, 28). These mechanistic findings provide partial evidence supporting the anti-cancer potential of mangos, which may warrant further investigation.

The present study has several strengths including the robust transcriptomic data collection and analysis procedures as well as the novelty of the approach, with the clustering analysis. We acknowledge that the use of clustering for the identification of hidden structures in data require extensive validation and is sensitive to the selection of input parameters. Herein, we tried to perform a comprehensive analysis in order to obtain relatively stable clusters that were finally able to answer biological questions that were initially masked. In any case, in view of the outcomes provided by such kind of clustering method, we cannot rule out that participants could be separated according to several dimensions of response, illustrated herein as distinct gene profiles. These additional layers of complexity may, however, be detrimental to classification performance (Supplementary Figure 6). Further works focused on improving algorithm and feature selection to enhance classification performance are ongoing in the field and have been extensively reviewed recently (29), revealing the complexity of classifying subjects by using gene expression profiles. Moreover, the nature of the intervention, involving mango pulp rather than mango in extracted/dehydrated/supplement form, is also novel. While the small sample size was a limitation of the study, in an effort to overcome the relatively low number of participants, the multilevel variation of the discriminant algorithms used in the present study was applied, which allowed to exploit the paired structure of the data obtained before and after the intervention in the same group of participants. Other limitations included the exploratory nature and the non-randomized design of the study, which limits our ability to establish cause and effect relationships about the impact of mango consumption on human health. The observed effects could have been associated with various confounding factors. However, the fact that the individuals enrolled in the project were subjected to a 2-week run-in period during which they were instructed to limit mango consumption and avoid significant variations in their health habits (diet, physical activity, etc.), somewhat isolates mango supplementation as the major dietary change of participants during the intervention. Still, our observations must be considered as exploratory to some extent and will require to be validated in future studies using randomized designs and select outcomes based on the identified metabolic pathways that were affected in the group of responders in the present study. Metabolic pathways related to blood pressure and glucose-insulin homeostasis may warrant more thorough investigation since the present study demonstrated that these metabolic parameters were improved following 8-week mango consumption.

## Conclusion

The results of the present study provide insights into the variable responses in gene expression and cardiometabolic clinical outcomes resulting from mango consumption. These results can be used to generate hypotheses for future studies exploring the impact of mango pulp on human health.

## Data availability statement

The original contributions presented in the study are publicly available. This data can be found here: http://www.ncbi.nlm.nih.gov/bioproject/865862.

## Ethics statement

This study was reviewed and approved by the Ethics Committee of Université Laval. All participants provided their written informed consent to participate in this study.

## Author contributions

JK conducted the statistical analyses on the clinical outcomes, prepared the first draft of the manuscript, and revised subsequent drafts. JT-M conducted the transcriptomic analyses. MK and VG coordinated the study. CC, GP, AM, and M-CV designed the study. CC oversaw all study activities. PC provided medical supervision. All authors reviewed/revised and approved the final manuscript.
