# Supplementary material for: Gene expression signatures and cardiometabolic outcomes following 8-week mango consumption in individuals with overweight/obesity
Source: Front Nutr. 2022 Aug 11;9:918844. doi: 10.3389/fnut.2022.918844 (PMC9407242; doi:10.3389/fnut.2022.918844)
Supplement: Supplementary file 1 [file Data_Sheet_1.docx]

**Gene expression signatures and cardiometabolic outcomes following 8-week mango consumption in individuals with overweight/obesity**

**Justine Keathley^1,2^, Juan de Toro-Martín^1,2^, Michèle Kearney^1,2^, Véronique Garneau^1,2^, Geneviève Pilon^1,3^, Patrick Couture^1,4^, André Marette^1,3^, Marie-Claude Vohl^1,2^, Charles Couillard^1,2*^**

^1^ Centre Nutrition, santé et société (NUTRISS)-Institut sur la nutrition et les aliments fonctionnels (INAF), Université Laval, Québec, QC G1V 0A6, Canada.

^2^ School of Nutrition, Université Laval, Québec, QC G1V 0A6, Canada.

^3^ Québec Heart and Lung Institute (IUCPQ) Research Center, 2725 Québec, QC G1V 4G5, Canada.

^4^ Endocrinology and Nephrology Unit, CHU de Quebec Research Center, Québec, QC G1V 4G2, Canada

*** Correspondance:**

Charles Couillard, PhD

charles.couillard@fsaa.ulaval.ca

**Supplementary Material**

**A B**

**Figure S1**. Outlier detection based on PCA components.

**A B**

**Figure S2.** Classification performance and explained variance from the PLS-DA model.

**A B**

**Figure S3**. Classification performance and variable selection from the sPLS-DA model.

**A B**

**Figure S4.** Pathway enrichment analysis with sPLS-DA components.

**A B**


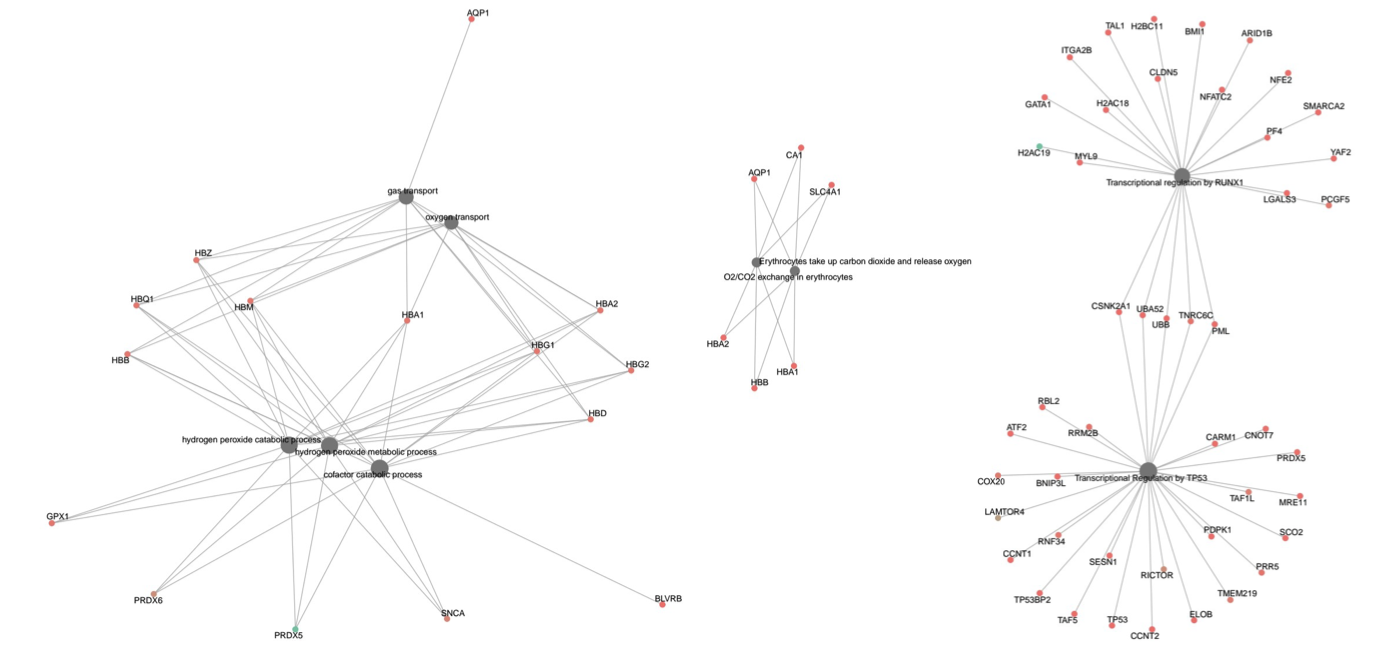


**Figure S5.** Pathway enrichment analysis with differentially regulated genes following mango consumption.

**A B**

**Figure S6.** Performance of a sPLS-DA model with six clusters based on two distinct responses.

**Table S1.** Top 100 up-regulated and down-regulated genes in the group of responders following the intervention.

| **RefSeq** | **FC** | **Gene symbol** | **Gene name** |
| --- | --- | --- | --- |
| **Up-regulated** |  |  |  |
| NM_183419 | 2.8 | *RNF19A* | Ring finger protein 19A, RBR E3 ubiquitin protein ligase |
| NM_001308195 | 2.8 | *SIMC1* | SUMO interacting motifs containing 1 |
| NM_173216 | 2.6 | *ST6GAL1* | ST6 beta-galactoside alpha-2,6-sialyltransferase 1 |
| NM_003774 | 2.5 | *GALNT4* | Polypeptide N-acetylgalactosaminyltransferase 4 |
| NM_001320967 | 2.5 | *HDLBP* | High density lipoprotein binding protein |
| NM_001270508 | 2.5 | *TNFAIP3* | TNF alpha induced protein 3 |
| NM_003455 | 2.5 | *ZNF202* | Zinc finger protein 202 |
| NR_145780 | 2.4 | *SNORD159* | Small nucleolar RNA, C/D box 159 |
| NM_015132 | 2.4 | *SNX13* | Sorting nexin 13 |
| NM_001318889 | 2.4 | *CD96* | CD96 molecule |
| NM_001278677 | 2.4 | *ZNF254* | Zinc finger protein 254 |
| NM_006595 | 2.3 | *API5* | Apoptosis inhibitor 5 |
| NM_001306093 | 2.3 | *ZADH2* | Zinc binding alcohol dehydrogenase domain containing 2 |
| NM_001278055 | 2.3 | *SACS* | Sacsin molecular chaperone |
| NM_001286795 | 2.3 | *SPATA13* | Spermatogenesis associated 13 |
| NM_001353165 | 2.3 | *ZMYM2* | Zinc finger MYM-type containing 2 |
| NM_006954 | 2.2 | *ZNF33A* | Zinc finger protein 33A |
| NM_012340 | 2.2 | *NFATC2* | Nuclear factor of activated T cells 2 |
| NM_001240 | 2.2 | *CCNT1* | Cyclin T1 |
| NM_001880 | 2.2 | *ATF2* | Activating transcription factor 2 |
| NM_001159920 | 2.2 | *FLT1* | Fms related tyrosine kinase 1 |
| NM_015210 | 2.2 | *MTCL1* | Microtubule crosslinking factor 1 |
| NR_152407 | 2.2 | *CUTALP* | Cuta divalent cation tolerance homolog-like, pseudogene |
| NM_001025107 | 2.2 | *ADAR* | Adenosine deaminase RNA specific |
| NM_144720 | 2.2 | *JAKMIP1* | Janus kinase and microtubule interacting protein 1 |
| NM_054026 | 2.2 | *CNOT7* | CCR4-NOT transcription complex subunit 7 |
| NM_001098524 | 2.2 | *NIBAN3* | Niban apoptosis regulator 3 |
| NM_004227 | 2.2 | *CYTH3* | Cytohesin 3 |
| NM_004655 | 2.2 | *AXIN2* | Axin 2 |
| NM_001367721 | 2.2 | *CASK* | Calcium/calmodulin dependent serine protein kinase |
| NM_016370 | 2.1 | *RAB9B* | RAB9B, member RAS oncogene family |
| NM_001282468 | 2.1 | *GOLGA8M* | Golgin A8 family member M |
| NM_015137 | 2.1 | *EFR3A* | EFR3 homolog A |
| NM_001145794 | 2.0 | *ANTXR2* | ANTXR cell adhesion molecule 2 |
| NM_177972 | 2.0 | *TUB* | TUB bipartite transcription factor |
| NM_005591 | 2.0 | *MRE11* | MRE11 homolog, double strand break repair nuclease |
| NM_001362914 | 2.0 | *DDHD2* | DDHD domain containing 2 |
| NR_159942 | 2.0 | *TRNT1* | Trna nucleotidyl transferase 1 |
| NM_173630 | 2.0 | *RTTN* | Rotatin |
| NM_018155 | 2.0 | *SLC25A36* | Solute carrier family 25 member 36 |
| NM_001127362 | 2.0 | *NR2C1* | Nuclear receptor subfamily 2 group C member 1 |
| NM_001322892 | 2.0 | *ABLIM1* | Actin binding LIM protein 1 |
| NM_001001671 | 2.0 | *MAP3K15* | Mitogen-activated protein kinase kinase kinase 15 |
| NM_001160243 | 2.0 | *RPAIN* | RPA interacting protein |
| NM_001304960 | 2.0 | *CCDC92* | Coiled-coil domain containing 92 |
| NM_001130864 | 2.0 | *PWWP2A* | PWWP domain containing 2A |
| NM_213648 | 2.0 | *TCF7* | Transcription factor 7 |
| NR_160436 | 2.0 | *HDAC7* | Histone deacetylase 7 |
| NM_001345875 | 1.9 | *PPIP5K2* | Diphosphoinositol pentakisphosphate kinase 2 |
| NM_144664 | 1.9 | *FAM76B* | Family with sequence similarity 76 member B |
| NM_032380 | 1.9 | *GFM2* | G elongation factor mitochondrial 2 |
| NR_135077 | 1.9 | *CSGALNACT2* | Chondroitin sulfate N-acetylgalactosaminyltransferase 2 |
| NM_001166006 | 1.9 | *EPB41* | Erythrocyte membrane protein band -4.1 |
| NR_039946 | 1.9 | *MIR4785* | Microrna 4785 |
| NM_001330700 | 1.9 | *TOP2B* | DNA topoisomerase II beta |
| NM_000410 | 1.9 | *HFE* | Homeostatic iron regulator |
| NM_080597 | 1.9 | *OSBPL1A* | Oxysterol binding protein like 1A |
| NM_001020658 | 1.9 | *PUM1* | Pumilio RNA binding family member 1 |
| NR_037597 | 1.9 | *LOC442028* | Uncharacterized LOC442028 |
| NM_152758 | 1.9 | *YTHDF3* | YTH N6-methyladenosine RNA binding protein 3 |
| NM_001282696 | 1.9 | *FAM107B* | Family with sequence similarity 107 member B |
| NM_005611 | 1.9 | *RBL2* | RB transcriptional corepressor like 2 |
| NR_164156 | 1.9 | *LOC101929950* | Puromycin-sensitive aminopeptidase-like protein |
| NM_001135663 | 1.9 | *RAB29* | RAB29, member RAS oncogene family |
| NM_001286051 | 1.9 | *FAM168A* | Family with sequence similarity 168 member A |
| NM_001080491 | 1.9 | *USP6NL* | USP6 N-terminal like |
| NM_014350 | 1.9 | *TNFAIP8* | TNF alpha induced protein 8 |
| NM_178313 | 1.9 | *SPTBN1* | Spectrin beta, non-erythrocytic 1 |
| NM_002907 | 1.9 | *RECQL* | Recq like helicase |
| NR_144468 | 1.9 | *TDRD6* | Tudor domain containing 6 |
| NM_182948 | 1.9 | *PRKACB* | Protein kinase camp-activated catalytic subunit beta |
| NR_138424 | 1.9 | *MIGA2* | Mitoguardin 2 |
| NR_161435 | 1.9 | *SNAPC3* | Small nuclear RNA activating complex polypeptide 3 |
| NM_032145 | 1.9 | *FBXO30* | F-box protein 30 |
| NM_021038 | 1.8 | *MBNL1* | Muscleblind like splicing regulator 1 |
| NM_001362746 | 1.8 | *REPIN1* | Replication initiator 1 |
| NM_181523 | 1.8 | *PIK3R1* | Phosphoinositide-3-kinase regulatory subunit 1 |
| NM_017941 | 1.8 | *C17orf80* | Chromosome 17 open reading frame 80 |
| NM_001105549 | 1.8 | *ZNF83* | Zinc finger protein 83 |
| NM_006060 | 1.8 | *IKZF1* | IKAROS family zinc finger 1 |
| NR_033302 | 1.8 | *DHX9* | Dexh-box helicase 9 |
| NR_036432 | 1.8 | *HERC2P3* | Hect domain and RLD 2 pseudogene 3 |
| NR_045553 | 1.8 | *THBS3* | Thrombospondin 3 |
| NM_001134232 | 1.8 | *TMEM106B* | Transmembrane protein 106B |
| NM_001244072 | 1.8 | *GIMAP6* | Gtpase, IMAP family member 6 |
| NM_006390 | 1.8 | *IPO8* | Importin 8 |
| NM_005238 | 1.8 | *ETS1* | ETS proto-oncogene 1, transcription factor |
| NM_015713 | 1.8 | *RRM2B* | Ribonucleotide reductase regulatory TP53 inducible subunit M2B |
| NM_001348413 | 1.8 | *NEK4* | NIMA related kinase 4 |
| NM_001991 | 1.8 | *EZH1* | Enhancer of zeste 1 polycomb repressive complex 2 subunit |
| NM_207122 | 1.8 | *EXT2* | Exostosin glycosyltransferase 2 |
| NM_001351517 | 1.8 | *TIA1* | TIA1 cytotoxic granule associated RNA binding protein |
| NM_152866 | 1.8 | *MS4A1* | Membrane spanning 4-domains A1 |
| NM_018976 | 1.8 | *SLC38A2* | Solute carrier family 38 member 2 |
| NM_002838 | 1.8 | *PTPRC* | Protein tyrosine phosphatase receptor type C |
| NM_001252124 | 1.8 | *PASK* | PAS domain containing serine/threonine kinase |
| NR_104295 | 1.8 | *CRELD2* | Cysteine rich with EGF like domains 2 |
| NM_006603 | 1.8 | *STAG2* | Stromal antigen 2 |
| NR_144517 | 1.8 | *LOC100996724* | Phosphodiesterase 4D interacting protein-like |
| NM_005734 | 1.8 | *HIPK3* | Homeodomain interacting protein kinase 3 |
| **Down-regulated** |  |  |  |
| NM_001256847 | -4.8 | *ABR* | ABR activator of rhogef and gtpase |
| NM_001287602 | -4.6 | *ARHGAP30* | Rho gtpase activating protein 30 |
| NM_001013253 | -3.9 | *LSP1* | Lymphocyte specific protein 1 |
| NR_102701 | -3.9 | *LINC00871* | Long intergenic non-protein coding RNA 871 |
| NM_001127615 | -3.7 | *ENOX1* | Ecto-NOX disulfide-thiol exchanger 1 |
| NR_134665 | -3.7 | *LINC02482* | Long intergenic non-protein coding RNA 2482 |
| NR_146114 | -3.5 | *VPS8* | VPS8 subunit of CORVET complex |
| NM_015070 | -3.1 | *ZC3H13* | Zinc finger CCCH-type containing 13 |
| NR_131012 | -3.1 | *NEAT1* | Nuclear paraspeckle assembly transcript 1 |
| NR_026731 | -3.0 | *LINC01551* | Long intergenic non-protein coding RNA 1551 |
| NR_046752 | -3.0 | *CADM2-AS2* | CADM2 antisense RNA 2 |
| NM_001190438 | -3.9 | *NCOR1* | Nuclear receptor corepressor 1 |
| NR_104146 | -3.9 | *LINC01206* | Long intergenic non-protein coding RNA 1206 |
| NM_177405 | -3.9 | *ADA2* | Adenosine deaminase 2 |
| NM_001145176 | -3.9 | *SHISA7* | Shisa family member 7 |
| NR_146120 | -3.8 | *RNA5-8SN4* | RNA, 5.8S ribosomal N4 |
| NM_016612 | -3.8 | *SLC25A37* | Solute carrier family 25 member 37 |
| NM_001271686 | -3.7 | *RAB3IL1* | RAB3A interacting protein like 1 |
| NM_001037967 | -3.6 | *ALAS2* | 5'-aminolevulinate synthase 2 |
| NR_003542 | -3.6 | *SLED1* | Proteoglycan 3, pro eosinophil major basic protein 2 pseudogene |
| NR_046851 | -3.6 | *ZNRF3-AS1* | ZNRF3 antisense RNA 1 |
| NM_001128831 | -3.6 | *CA1* | Carbonic anhydrase 1 |
| NR_037852 | -3.5 | *DDX39B* | Dexd-box helicase 39B |
| NR_047498 | -3.5 | *LINC00853* | Long intergenic non-protein coding RNA 853 |
| NM_001112812 | -3.5 | *GRIA4* | Glutamate ionotropic receptor AMPA type subunit 4 |
| NM_138456 | -3.5 | *BATF2* | Basic leucine zipper ATF-like transcription factor 2 |
| NM_181539 | -3.5 | *KRT26* | Keratin 26 |
| NM_001077262 | -3.5 | *UBXN11* | UBX domain protein 11 |
| NR_027231 | -3.5 | *LINC00685* | Long intergenic non-protein coding RNA 685 |
| NM_003944 | -3.5 | *SELENBP1* | Selenium binding protein 1 |
| NM_001318221 | -3.4 | *AHSP* | Alpha hemoglobin stabilizing protein |
| NM_001128830 | -3.4 | *CA1* | Carbonic anhydrase 1 |
| NM_002036 | -3.4 | *ACKR1* | Atypical chemokine receptor 1 (Duffy blood group) |
| NM_001370737 | -3.4 | *TMCO5A* | Transmembrane and coiled-coil domains 5A |
| NM_153809 | -3.4 | *TAF1L* | TATA-box binding protein associated factor 1 like |
| NM_001288744 | -3.4 | *CMTM5* | CKLF like MARVEL transmembrane domain containing 5 |
| NR_134624 | -3.4 | *LOC105369595* | Uncharacterized LOC105369595 |
| NR_047499 | -3.4 | *LINC00570* | Long intergenic non-protein coding RNA 570 |
| NR_027782 | -3.4 | *PLEKHM1* | Pleckstrin homology and RUN domain containing M1 |
| NM_016633 | -3.4 | *AHSP* | Alpha hemoglobin stabilizing protein |
| NM_006121 | -3.4 | *KRT1* | Keratin 1 |
| NM_018996 | -3.3 | *TNRC6C* | Trinucleotide repeat containing adaptor 6C |
| NM_000032 | -3.3 | *ALAS2* | 5'-aminolevulinate synthase 2 |
| NM_000559 | -3.3 | *HBG1* | Hemoglobin subunit gamma 1 |
| NM_001033024 | -3.3 | *FBXO7* | F-box protein 7 |
| NR_037612 | -3.3 | *SEPT5-GP1BB* | SEPT5-GP1BB readthrough |
| NR_120526 | -3.3 | *LOC100506675* | Uncharacterized LOC100506675 |
| NM_001357734 | -3.3 | *EIF2S3B* | Eukaryotic translation initiation factor 2 subunit gamma B |
| NR_046511 | -3.3 | *MED4-AS1* | MED4 antisense RNA 1 |
| NM_001258326 | -3.3 | *SFI1* | SFI1 centrin binding protein |
| NM_000519 | -3.3 | *HBD* | Hemoglobin subunit delta |
| NM_001318236 | -3.3 | *LAMTOR4* | Late endosomal/lysosomal adaptor, MAPK and MTOR activator 4 |
| NM_001003938 | -3.3 | *HBM* | Hemoglobin subunit mu |
| NM_001207035 | -3.3 | *ETV7* | ETS variant transcription factor 7 |
| NM_001287580 | -3.3 | *RBKS* | Ribokinase |
| NM_001037968 | -3.3 | *ALAS2* | 5'-aminolevulinate synthase 2 |
| NM_000184 | -3.3 | *HBG2* | Hemoglobin subunit gamma 2 |
| NM_005331 | -3.3 | *HBQ1* | Hemoglobin subunit theta 1 |
| NM_006778 | -3.3 | *TRIM10* | Tripartite motif containing 10 |
| NM_001354943 | -3.3 | *CUL4A* | Cullin 4A |
| NM_001042461 | -3.3 | *TRAPPC5* | Trafficking protein particle complex 5 |
| NM_138442 | -3.3 | *CCDC124* | Coiled-coil domain containing 124 |
| NM_001142806 | -3.3 | *SLC6A8* | Solute carrier family 6 member 8 |
| NM_001308023 | -3.3 | *TNS1* | Tensin 1 |
| NR_039656 | -3.3 | *MIR4451* | Microrna 4451 |
| NR_026911 | -3.3 | *RPL21P28* | Ribosomal protein L21 pseudogene 28 |
| NM_080658 | -3.3 | *ACY3* | Aminoacylase 3 |
| NM_175710 | -3.3 | *CR1L* | Complement c3b/C4b receptor 1 like |
| NM_001244962 | -3.3 | *FRMD3* | FERM domain containing 3 |
| NR_033850 | -3.3 | *WDR11-AS1* | WDR11 antisense RNA 1 |
| NM_001291824 | -3.2 | *GRAP2* | GRB2 related adaptor protein 2 |
| NM_000558 | -3.2 | *HBA1* | Hemoglobin subunit alpha 1 |
| NR_002319 | -3.2 | *PIPSL* | PIP5K1A and PSMD4 like (pseudogene) |
| NR_051985 | -3.2 | *DDX11L9* | DEAD/H-box helicase 11 like 9 |
| NM_002100 | -3.2 | *GYPB* | Glycophorin B (MNS blood group) |
| NM_152718 | -3.2 | *VWCE* | Von Willebrand factor C and EGF domains |
| NM_002476 | -3.2 | *MYL4* | Myosin light chain 4 |
| NM_006563 | -3.2 | *KLF1* | Kruppel like factor 1 |
| NM_000517 | -3.2 | *HBA2* | Hemoglobin subunit alpha 2 |
| NM_030885 | -3.2 | *MAP4* | Microtubule associated protein 4 |
| NR_002221 | -3.2 | *DPRXP4* | Divergent-paired related homeobox pseudogene 4 |
| NM_013401 | -3.2 | *RAB3IL1* | RAB3A interacting protein like 1 |
| NM_014869 | -3.2 | *IQSEC1* | IQ motif and Sec7 domain arfgef 1 |
| NM_001349109 | -3.2 | *ST6GALNAC3* | ST6 N-acetylgalactosaminide alpha-2,6-sialyltransferase 3 |
| NM_001290403 | -3.2 | *TAL1* | TAL bhlh transcription factor 1, erythroid differentiation factor |
| NM_001172673 | -3.2 | *CELF5* | CUGBP Elav-like family member 5 |
| NM_001114134 | -3.2 | *EPB42* | Erythrocyte membrane protein band -4.2 |
| NM_000119 | -3.2 | *EPB42* | Erythrocyte membrane protein band -4.2 |
| NM_001130985 | -3.2 | *DYSF* | Dysferlin |
| NM_000342 | -3.2 | *SLC4A1* | Solute carrier family 4 member 1 (Diego blood group) |
| NM_016016 | -3.1 | *SLC25A39* | Solute carrier family 25 member 39 |
| NM_001008778 | -3.1 | *SPDYC* | Speedy/RINGO cell cycle regulator family member C |
| NM_032498 | -3.1 | *RHOXF2* | Rhox homeobox family member 2 |
| NM_020957 | -3.1 | *PCDHB16* | Protocadherin beta 16 |
| NR_027133 | -3.1 | *LINC00924* | Long intergenic non-protein coding RNA 924 |
| NM_001271606 | -3.1 | *BASP1* | Brain abundant membrane attached signal protein 1 |
| NM_001007563 | -3.1 | *IGFBPL1* | Insulin like growth factor binding protein like 1 |
| NR_146626 | -3.1 | *LINC00537* | Long intergenic non-protein coding RNA 537 |
| NM_015865 | -3.1 | *SLC14A1* | Solute carrier family 14 member 1 (Kidd blood group) |

Differentially expressed genes at FDR-adjusted p-value < 0.05. RefSeq, reference sequence accession number. Positive and negative fold changes (FC) stand for up- and down-regulation following the intervention, respectively.
